# Diquat Herbicide Organophosphate Poisoning and Multi-Organ Failure: A Case Report

**DOI:** 10.7759/cureus.27241

**Published:** 2022-07-25

**Authors:** Daniel M Aloise, Adam Memon, Ana Zaldivar

**Affiliations:** 1 Department of Translational Medicine, Herbert Wertheim College of Medicine, Florida International University, Miami, USA; 2 Emergency Department, Baptist Memorial Hospital, Miami, USA

**Keywords:** poison ingestion, paraquat, reactive oxygen species, toxicology, multiorgan failure, organophosphate poisoning, herbicide, diquat

## Abstract

A 63-year-old male landscaper presented to our emergency department (ED) following unintentional ingestion of an herbicide including diquat, a highly lethal toxin. Poison control was consulted, and treatment was centered around emergency hemodialysis to mitigate the nephrotoxic effects of diquat. Unfortunately, our patient did not survive. Unlike most organophosphate poisonings, diquat facilitates the formation of catastrophic amounts of reactive oxygen species leading to lethal consequences for affected cells. The body’s most affected sites are the kidneys, liver, and lungs.

Our patient’s accidental diquat consumption illuminates the need for additional research to be done. This case report emphasizes the importance of identifying effective treatments for diquat and similar poisonings that lead to oxidative stress. We seek to describe this uncommon instance of accidental diquat ingestion, including the unique therapeutic regimen, and course of disease progression.

## Introduction

Diquat is a toxin that functions as an herbicide used to control terrestrial and aquatic vegetation, which is rarely encountered in the United States [1]. Diquat ingestion often manifests as organophosphate poisoning and multi-organ failure secondary to formation of reactive oxygen species [2]. Diquat’s toxic effects are due to its propensity to undergo a series of oxidation and reduction reactions, leading to the formation of superoxide anion radicals and hydrogen peroxide [2]. In large quantities, this can overwhelm the body’s protective detoxification methods, which utilize catalase and glutathione peroxidase [2]. Diquat most commonly targets the kidney, manifesting as tubular necrosis, followed by the lung, liver, brain, and heart [1]. There is no specific antidote for diquat poisoning, and multi-organ failure from diquat poisoning is relatively rare, especially with neurological involvement, with few cases reported in literature [1,3]. We seek to describe this uncommon instance of accidental diquat ingestion in our patient, including therapeutic regimens, pathophysiology, and course of disease progression characterized by multi-organ failure.

## Case presentation

A 63-year-old male presented to our emergency department approximately 90 minutes after ingestion of Tribune herbicide (Cygnet Enterprises Inc., Michigan, United States), containing the toxic chemical, diquat. This landscaper swallowed a “small gulp” of liquid from a sports drink bottle, not knowing that this bottle had been filled with herbicide. Instantly after ingestion, the patient realized based on the taste that he drank something other than a sports drink. He was soon informed it was herbicide he had mistakenly consumed yet was unsure if he should immediately go to the emergency department for treatment.

The patient soon experienced a burning sensation in his throat and sternal area, as well as nausea and vomiting, which led to his emergent presentation to the ED. The patient presented in moderate acute distress with severe epigastric discomfort with urinary incontinence, vomiting, diarrhea, and weakness of his lower extremities. The diarrhea was watery and neon green in color. The vomit was mostly clear liquid and did not contain blood. The patient lacked bronchorrhea, and his pupils were normal in size. He was hemodynamically stable on presentation, with a body mass index of 25.6 kg/m2, temperature of 36.6 degrees Celsius, heart rate of 74 beats per minute, respiratory rate of 18 breaths per minute, blood pressure of 149/86 mm Hg, and O2 saturation of 100% on room air. The patient displayed severe epigastric abdominal discomfort with diaphoresis and bilateral lower extremity weakness.

Poison control was immediately called, which stated the exceptionally high mortality associated with remote diquat ingestion, which, though rarely encountered, typically leads to multi-organ failure and death within days to weeks [2]. Recommendation was given by Poison control for immediate decontamination and emergent dialysis. Atropine was also recommended as needed should bronchorrhea arise, as well benzodiazepines as needed for seizure control. The nephrology department was consulted, and dialysis begun, along with plans to monitor creatinine every four to six hours. Laboratory reports on admission featured in Table 1 included an elevated lactate and white blood cell (WBC) count. Electrocardiogram on admission showed normal sinus rhythm with incomplete right bundle branch block.

**Table 1:** Patient laboratory findings on admission AST: aspartate transaminase

	Patient Value on Admission	Reference Range
Lactate	3.0 mmol/L	0.5 – 2.2 mmol/L
Creatinine	1.0 mg/dL	0.6 – 1.2 mg/dL
AST	29 U/L	12 – 38 U/L
White Blood Cells	14.7 x 10^9 ^/L	4.5 – 11.0 x 10^9^/L

Following thorough decontamination of the patient and his clothes, a central line was placed for dialysis access. This was done on the patient’s right internal jugular vein, as the femoral site was compromised by persistent diarrhea. The patient proceeded to undergo dialysis continuously for the next three days. Notably, despite displaying many classic characteristics of organophosphate poisoning, including urination, diarrhea and gastric emesis, atropine was not administered, as the patient did not develop bronchorrhea.

Over the next 24 hours our patient developed worsening kidney failure. We observed rising creatinine and aspartate transaminase (AST), while his already elevated lactate and WBC count continued to rise. Table 2 shows relevant laboratory values for our patient 24 hours from time of admission. The patient again received dialysis on hospital day two, and following gastrointestinal (GI) consult, the patient was also started on acetylcysteine and dexamethasone.

**Table 2:** Patient laboratory findings 24 hours from admission AST: aspartate transaminase

	24 Hours Post-Admission	Reference Range
Lactate	4.4 mmol/L	0.5 – 2.2 mmol/L
Creatinine	1.5 mg/dL	0.6 – 1.2 mg/dL
AST	139 U/L	12 – 38 U/L
White Blood Cells	21.1 x 10^9 ^/L	4.5 – 11.0 x 10^9^/L

A code rescue was called on hospital day three for new-onset atrial fibrillation with rapid ventricular response, and patient was subsequently started on a diltiazem infusion. It was then noted that the patient became intermittently confused and began pulling out intravenous (IV) lines, with his new-onset confusion suspected to be secondary to the setting of acute toxic metabolic encephalopathy. The patient was noted to have post-ictal rigidity, and subsequently was transferred to the intensive care unit (ICU), where he received dialysis and IV fluids. The neurology department was consulted, and an electroencephalogram (EEG) was conducted. Video EEG revealed that the patient suffered from tonic-clonic generalized seizures, and his EEG findings were indicative of bilateral cerebral dysfunction. The patient was intubated on his transfer to the ICU.

On day four of hospitalization, the patient was noted to have new onset dilated pupils and was taken to receive a CT scan of his brain, which showed diffuse cerebral edema and toxic encephalopathy with cerebellar tonsillar herniation and mild hydrocephalus. At this time the patient was noted to be hypotensive despite use of vasopressors. He was continued on midazolam, which had been held for the neurological exam. The patient was evaluated by neurosurgery who recommended no neurosurgical intervention at this time, as any such surgical option was deemed futile in changing the outcome for this patient. At this time the patient was receiving hypertonic saline and was seen by neurology, at which point the patient was noted to have progressive neurological deterioration accompanied by loss of brainstem reflexes with occasional spontaneous respirations. Following discussion with the patient’s family, he was transitioned to receive comfort measures only. He was pronounced dead on day four of hospitalization, secondary to cardiac arrest.

## Discussion

Organophosphate poisoning is well-described toxic ingestion with many classic characteristics and standardized treatment regimens. These traditional signs include the often mentioned “DUMBBBELS” mnemonic, featuring defecation, urination, miosis, bronchorrhea, bradycardia, bronchospasm, emesis, lacrimation, and salivation [4]. First-line treatment includes atropine, which competes with acetylcholine at muscarinic receptors to prevent and/or inhibit cholinergic crisis [4]. Additionally, pralidoxime is often used as an adjunct therapy alongside atropine to combat muscle weakness, including respiratory failure. Pralidoxime acts as a competitive inhibitor of acetylcholine at nicotinic sites to regulate neuromuscular dysfunction [4].

Diquat consumption, in contrast with organophosphate poisoning, is an extremely rare occurrence with very few cases described in great detail [2]. Worldwide, there are approximately 3,000,000 organophosphate exposures annually with about 300,000 deaths, while in 2008 in the United States there were an estimated 800 exposures and 15 deaths [4]. This is in stark comparison to diquat consumption, as from 1968 to 1999 there were only 30 cases documented in the U.S. with a 43% mortality [2].

The lethal dose of diquat for humans has been found to be just 6-12 g [4]. The Tribune herbicide that was ingested by our patient contains 2 lbs of diquat per gallon, which equates to approximately 24g/100mL. This indicates that a lethal dose of diquat (6 g) may be consumed with ingestion of approximately 25 mL of herbicide. A study performed by Hitchings et al in 2013 suggests the average “small mouthful” for toxic ingestion is about 43 mL, which would equate to a supra-lethal dose of about 10g of diquat in the case of our patient, who reported taking a “small gulp” from the bottle [5]. For comparison, a “large gulp” was estimated to be 77 mL, which would contain more than 18 grams of diquat [5]. Studies have shown that fulminant poisoning (> 12 g diquat) is characterized by multiple organ failures and death in nearly all cases within 24-48 hours [5]. We note these specific distinctions of the volume ingested to emphasize the potency of diquat and the urgency for emergent treatment that even small ingestion necessitates.

The initial signs of diquat poisoning are centered around organophosphate poisoning, which is due to the inhibition of acetylcholinesterase, the enzyme responsible for breaking down acetylcholine. An overabundance of this molecule manifests as cholinergic toxicity manifesting as muscarinic and/or nicotinic toxicity. Our patient displayed signs of muscarinic over-activation, including diarrhea, diaphoresis, urination, and emesis. He also exhibited signs of nicotinic toxicity, as evidenced by his lower extremity weakness.

**Figure 1:**
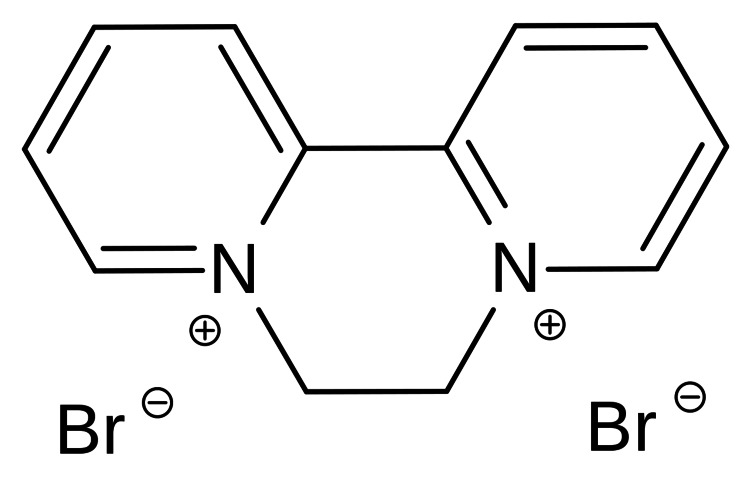
Chemical structure of diquat Diquat is the organic cation formed formally by addition of an ethylene bridge between the nitrogen atoms of 2,2'-bipyridine. Image source: Wikimedia, permitted for re-use and does not require specific permission, as per Wikimedia commons guidelines [6].

**Figure 2:**
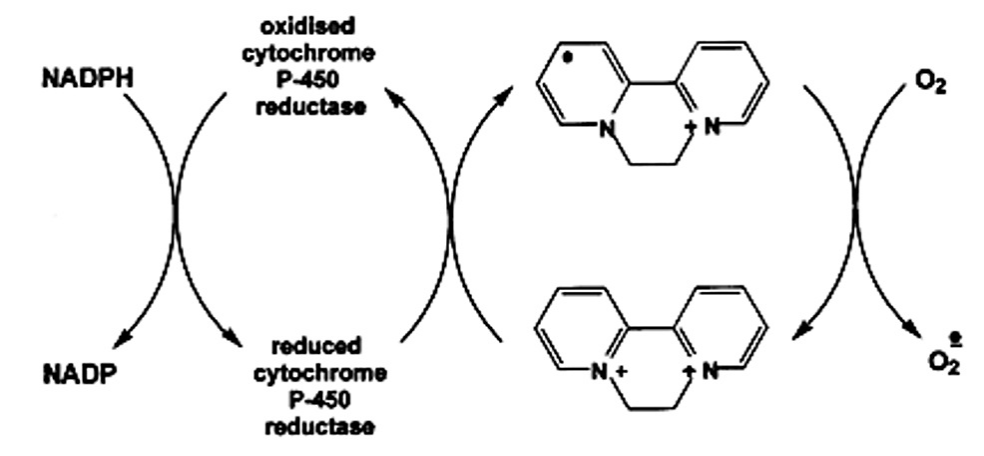
Redox cycling of diquat Using NADPH and cytochrome P450 reductase, diquat forms a highly unstable radical. This radical transfers an electron to molecular oxygen to form a superoxide anion radical, which is a highly reactive species. With cyclic reduction-oxidation reactions, the toxic action of diquat leads to mass formation of ROS and depletion of reduced NADPH. Image source: Taylor & Francis Group from an article published by Jones et al., with its rights purchased for re-use in this journal [2].

The mortality of diquat is most commonly due to the formation of reactive oxygen species (ROS), which can lead to multi-organ failure, as displayed in our patient [4]. Figure 1 displays the chemical structure of diquat. Diquat is a potent redox cycler with its toxic effects dependent on its ability to undergo a single electron addition to form a free radical [2]. Figure 2 demonstrates the highly unstable diquat radical that is formed via nicotinamide adenine dinucleotide phosphate (NADPH) and cytochrome P450 reductase. This radical transfers an electron to molecular oxygen to form a superoxide anion radical, which is a highly reactive species [2].

**Figure 3:**
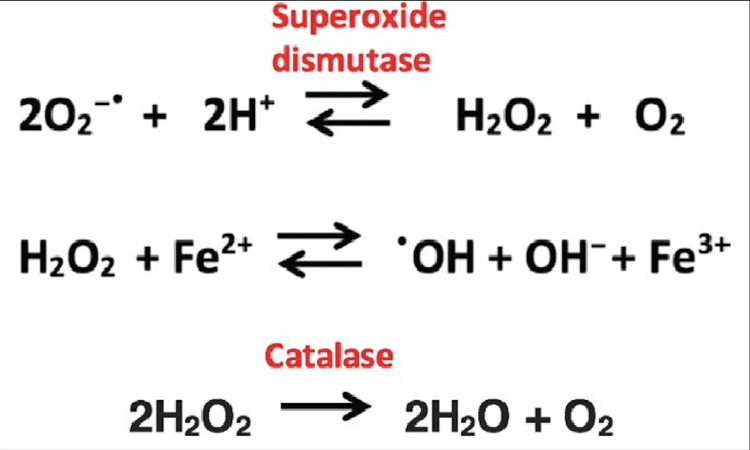
Dismutation of superoxide anion radical (1st) and hydroxyl radical formation (2nd) Superoxide anion radicals (O2-·) produced from redox cycling of diquat react with each other to form hydrogen peroxide (H2O2) and molecular oxygen (O2). Second reaction depicts formation of the hydroxyl radical and ferric iron (Fe3+) from hydrogen peroxide and ferrous iron (Fe2+). Catalase can detoxify hydrogen peroxide to form water (H2O) and oxygen, as shown in the third and final reaction. Image source: Tripaydonis et al.  [7]. Figures from this article are free to be copied, redistributed, and re-used, as per licensure agreement located at https://creativecommons.org/licenses/by-nc/4.0/.

Diquat is continuously cycled in this process of oxidation and reduction, as the superoxide anion radicals produced from the redox cycling of diquat react with each other to form hydrogen peroxide and molecular oxygen [2]. This reaction may occur either via the enzyme superoxide dismutase, as shown in Figure 3, or spontaneously [7]. Hydrogen peroxide (H2O2) can then go on to react with ferrous iron (Fe2+) to form ferric iron (Fe3+) and an even more powerful oxidant hydroxyl radical (·OH) [8]. Under normal physiological conditions, our body can detoxify hydrogen peroxide via the enzymes catalase and glutathione peroxidase, however, our protective mechanisms can become overwhelmed, leading to detrimental consequences for affected cells [2].

The most affected sites are the kidneys, liver, and lungs, with acute tubular necrosis being pathognomonic [4]. Consistent with the aforementioned findings of diquat poisoning, our patient exhibited rapid renal decline as well as liver impairment. This is evidenced by his creatinine elevation from 1 to 5 within four days of diquat ingestion and a five-fold increase in AST within just 24 hours. Diquat is a hydrophilic substance whose ingestion leads to oxidative stress via the production of ROS, which typically spares the brain and spinal cord [8]. However, a case report by Xing et al in 2020 describes a rare case of diquat ingestion manifesting as central pontine myelinolysis [1]. Similarly, our patient exhibited neural manifestations. He experienced tonic-clonic seizures with post-ictal rigidity and suffered from diffuse cerebral edema with toxic encephalopathy, cerebellar tonsillar herniation, and mild hydrocephalus.

Diquat is a highly caustic substance, which may cause severe and extensive mucosal damage to the mouth, esophagus, stomach, and small intestine. Postmortem studies have shown diffuse erosions and mucosal necrosis in the esophagus, stomach, and ileum [2]. Paralytic ileus is one notable complication that may develop within one to four days of ingestion; this ileus is hypothesized to be responsible for the sequestration of large amounts of fluid in the gut, ultimately leading to hypovolemic shock [2]. Nephrotoxicity with renal failure has been shown to develop as quickly as one hour to five days post-ingestion [2]. Renal failure is the result of two processes: reduced renal perfusion resulting from hypovolemia, and a direct toxic effect of diquat on the kidney characterized by acute tubular necrosis - this exact mechanism of action is not fully understood at this time [2]. Pulmonary findings include bronchopneumonia, characterized by radiological appearances of pulmonary infiltrates and exudates similar to that observed in acute respiratory distress syndrome (ARDS) [2]. Cardiotoxic effects have been reported in many cases of diquat ingestion. Several patients are noted to have developed atrial fibrillation and ventricular arrhythmias, with multiple patients ultimately succumbing to uncorrectable ventricular arrhythmias and cardiac arrest, as was the case in our patient [2]. Notably, the following features have been identified as incurring a poor prognosis: rapid onset of acute renal failure, ventricular arrhythmias, intestinal ileus, subsequent fluid sequestration, pulmonary complications requiring ventilation, and coma [2].

Currently, there is no antidote or effective treatment for diquat intoxication. Instead, treatment is focused on decreasing absorption and enhancing elimination [8]. The guidelines call for immediate decontamination and activated charcoal for gut detoxification within one hour of consumption [8]. Notably, activated charcoal would have been administered to our patient had he presented within one hour of diquat ingestion.

Atropine and pralidoxime may be utilized for symptomatic improvement of the initial cholinergic toxicity, while hemodialysis can aid with the elimination of diquat [8]. Various other treatments including antioxidants, corticosteroids, and benzodiazepines may be considered for symptomatic improvement. N-acetylcysteine and vitamin E have been utilized as an adjunct therapy, as antioxidant compounds can combat overwhelming amounts of ROS that deplete our body’s stores of glutathione [9].

While rare in occurrence in the United States, our patient’s tragic accidental consumption of diquat illustrates the need for additional research to be done in this area. According to a study by Fortenberry et al. in 2016, most deaths associated with paraquat and diquat were due to work-related ingestion; however, nearly a third of the deaths identified were due to unintentional ingestion, often the result of improper storage in beverage bottles, as was the case for our patient [10]. Our case report’s findings emphasize the importance of spreading awareness of the danger associated with diquat and promoting proper storage and handling of this toxic chemical, as there are currently no antidotes to its consumption. Additionally, this case highlights the significance of identifying effective treatments for diquat and similar poisonings that results in oxidative stress secondary to the formation of ROS. Such research may save lives in the United States, with implications expanding far beyond the boundaries of our country. Thousands of people may stand to benefit, including in countries such as China, where diquat is utilized routinely and poisonings are increasing since the banning of Paraquat (Syngenta AG, Basel, Switzerland) in 2016, a similar toxin previously used in herbicides [1].

## Conclusions

Our patient received urgent treatment at our emergency department after accidental diquat consumption. He exhibited many of the classic signs of organophosphate poisoning and subsequent multi-organ failure consistent with diquat poisoning. Despite urgent treatment following recommendations from poison control, he ultimately passed away on hospital day four secondary to cardiac arrest and multi-organ failure. By describing this rare instance of diquat poisoning, we hope to elicit additional awareness to avoid future accidental consumption and promote knowledge of therapeutic regimens and course of disease progression. Additionally, we hope to demonstrate the extreme potency of this chemical and the need for emergent treatment, as consumption of even the smallest amount can be lethal without timely use of charcoal. We hope this case report will lead to additional research on treatments for diquat poisoning and other related toxicities that lead to overwhelming oxidative stress.
